# Effect of Adjuvant Therapy on Oncologic Outcomes of Surgically Confirmed Stage I Uterine Carcinosarcoma: a Turkish Gynecologic Oncology Study

**DOI:** 10.4274/balkanmedj.galenos.2019.2018.12.75

**Published:** 2019-07-11

**Authors:** Günsu Kimyon Cömert, Osman Türkmen, Gökhan Boyraz, İbrahim Yalçın, Duygu Altın, Alper Karalök, Hanifi Şahin, Salih Taşkın, Derman Başaran, Zeliha Fırat Cuylan, Kazibe Koyuncu, Mehmet Coşkun Salman, Nejat Özgül, Mehmet Mutlu Meydanlı, Taner Turan, Fırat Ortaç, Kunter Yüce

**Affiliations:** 1 Department of Gynecologic Oncology, Ankara Etlik Zübeyde Hanım Women’s Diseases Training and Research Hospital, Ankara, Turkey; 2 Department of Gynecologic Oncology, Hacettepe University Faculty of Medicine, Ankara, Turkey; 3 Department of Gynecologic Oncology, University of Health Sciences, Ankara Zekai Tahir Burak Women’s Health Training and Research Hospital, Ankara, Turkey; 4 Department of Gynecologic Oncology, Ankara University School of Medicine, Ankara, Turkey

**Keywords:** Adjuvant, carcinosarcoma, chemotherapy, radiotherapy, uterine neoplasms

## Abstract

**Background:**

Uterine carcinosarcoma is rare neoplasm that mostly presents as metastatic disease. Stage is one of the most important prognostic factor, however, the management of the early stage uterine carcinosarcoma is still controversial.

**Aims:**

To evaluate prognostic factors, treatment options, and survival outcomes in patients with surgically approved stage I uterine carcinosarcoma.

**Study Design:**

Cross-sectional study.

**Methods:**

Data of 278 patients with uterine carcinosarcoma obtained from four gynecologic oncology centers were reviewed, and 70 patients with approved stage I uterine carcinosarcoma after comprehensive staging surgery were studied.

**Results:**

The median age of the entire cohort was 65 years (range; 39-82). All patients underwent both pelvic and paraaortic lymphadenectomy. Forty-one patients received adjuvant therapy. The median follow-up time was 24 months (range; 1-129). Nineteen (27.1%) patients had disease failure. The 3-year disease-free survival and cancer-specific survival of the entire cohort was 67% and 86%, respectively. In the univariate analysis, only age was significantly associated with disease-free survival (p=0.022). There was no statistical significance for disease-free survival between observation and receiving any type of adjuvant therapy following staging surgery. Advanced age (<75 vs ≥75 years) was the only independent prognostic factor for recurrence (hazard ratio: 3.8, 95% CI=1.10-13.14, p=0.035) in multivariate analysis. None of the factors were significantly associated with cancer-specific survival.

**Conclusion:**

Advanced age was the only independent factor for disease-free survival in stage I uterine carcinosarcoma. Performing any adjuvant therapy following comprehensive lymphadenectomy was not related to the improved survival of the stage I disease.

Uterine carcinosarcoma (UCS) is a rare uterine neoplasm, with an incidence of 3%-4% among all uterine malignancies (1). UCS is associated with high risk for metastatic disease at presentation, recurrence, and poor survival (1,2,3). Pathologic stage is the most important predictive factor for survival, but the recurrence rate is high, even in the early stages of the disease (2,4). The incidence of stages I and II disease is 35%-40% (5,6).

Definitive staging surgery that includes complete lymphadenectomy is recommended as maintenance treatment for early-stage UCS (1,6,7,8). Furthermore, the survival benefit of lymphadenectomy increases with the increasing number of lymph nodes removed (8). However, controversial results exist regarding both the necessity and type of adjuvant therapy in the presence of high quality lymphadenectomy (5,6,9,10,11). Adjuvant radiotherapy likely improves local control but has no significant effect on survival of early-stage UCS (11,12). Although utilization of adjuvant chemotherapy and chemo-radiotherapy has been investigated more frequently because of the tendency of distant recurrence in UCS, even with early-stage disease (6), the optimal postoperative management is still controversial in the early-stage.

Survival rates are lower in stage II UCS, but patients with cervical invasion have not been excluded in the majority of reports related to early-stage UCS (6,9,12,13,14). In addition, some of these studies have included patients who had no comprehensive surgical staging, including lymphadenectomy. Therefore, the main aim of the current investigation is to evaluate the prognostic factors, treatment options, and survival outcomes in patients with only surgically confirmed stage I UCS to minimize the factors that can affect survival.

## MATERIALS AND METHODS

The data of patients who underwent at least total abdominal hysterectomy and bilateral salpingo-oophorectomy, and whose definitive pathology report revealed UCS between January 1993 and March 2017, were obtained from four gynecologic oncology centers, retrospectively. All patients signed an informed consent that allows the participating institution to use their clinical data. Institutional review board approval was obtained before the study.

All surgeries were performed by gynecologic oncologists, and all pathologies were reported by pathologists specialized in gynecologic oncology at each institution. Records of a total of 278 patients who had a pathologic report of UCS were evaluated. The absence of comprehensive lymphadenectomy, having stage II and above disease, and presence of synchronized tumors were the exclusion criteria of the study. Finally, the study included 70 patients with surgically confirmed stage I UCS (25%).

Staging surgery standardly involves a total abdominal hysterectomy, bilateral salpingo-oophorectomy, systematic pelvic-paraaortic lymphadenectomy, omentectomy, and cytologic sampling. Lymphadenectomy was performed by skeletonizing both pelvic and paraaortic regions. The upper limit of paraaortic lymphadenectomy was the left renal vein. Patients were staged according to the International Federation of Gynecology and Obstetrics 2009 surgical staging criteria of UCS. Tumor size was defined as the largest diameter of the tumor. Adjuvant therapy was decided by the gynecologic oncology counsel, and adjuvant chemotherapy was categorized as a paclitaxel-based and non-paclitaxel-based chemotherapy.

Patients who had a complete clinical response to initial treatment were followed up quarterly for the first 2 years, semi-annually up to 5 years, and annually after that. A pelvic examination, abdominal-pelvic ultrasonography, and an annual chest X-ray, unless there was a clinical suspicion, were performed during the follow-up. Thoracic and/or abdominal tomography was used when needed. Patients whose routine evaluations during follow-up showed the absence of the disease in the first month after initial treatment but whose disease recurred was accepted as a recurrence. Progressive disease and recurrence were handled as a disease failure after initial therapy. Disease-free survival (DFS) was defined as the time from initial surgery to the failure of disease or last contact. The period from initial surgery to death, because of the disease, or the last visit was defined as cancer-specific survival (CSS). We defined recurrence distal to the pelvic inlet as pelvic recurrence, between the pelvic inlet and diaphragm as abdominal recurrence, and the rest of recurrences as extra-abdominal recurrence. Recurrence in the liver parenchyma, skin, and bone was accepted as extra-abdominal recurrence.

SPSS 17.0 (SPSS Inc., Chicago, IL, USA) was used for statistical analysis. Descriptive statistics were expressed as mean ± standard deviation or median (minimum-maximum) for continuous variables and number/percentage for categorical variables. Categorical variables were compared using the chi-square or Fisher’s exact test, as appropriate. The Kaplan–Meier method was used for the assessment of survival outcomes. Survival curves were compared using the log-rank test. Variables with a p value <0.25 in univariate analysis were selected to evaluate the correlation among variables. After determining the factors that were not inter-related, a model of recurrence was established for multivariate analysis. Multivariate analysis was performed using a Cox proportional hazards model. The level of statistical significance was set at p<0.05.

## RESULTS

The median age of the entire cohort was 65 years (range; 39-82 years). All patients underwent both pelvic and paraaortic lymphadenectomy. The median number of lymph nodes removed was 44 (range; 5-120). Fifty-nine percent of patients had stage IA disease. Lymphovascular space invasion was determined in 29% of patients. Forty-one (58.6%) patients received adjuvant therapy. The clinical-pathological findings are shown in Table 1.

Adjuvant therapy was performed as only chemotherapy in 22 patients, only radiotherapy in eight patients, and chemotherapy with radiotherapy in 11 patients. All patients in the paclitaxel-based chemotherapy group (n=22) received the paclitaxel and carboplatin protocol. Performed protocols for the non-paclitaxel chemotherapy group are detailed in Table 1. Stage distribution did not significantly differ between groups with and without adjuvant therapy (p=0.319).

The median follow-up was 24 months, ranging from 1 to 129 months. Nineteen (27.1%) patients had disease failure. Four patients had disease failure as progressive disease, and 15 patients experienced recurrence. Among patients with recurrence, recurrence localizations included the pelvic area in 33%, abdominal area in 40%, and the extra-abdominal area in 47%. Also, recurrences occurred that were only pelvic (13.3%), only abdominal (20%), and only extra-abdominal recurrences (26.6%). The most common involved organ in recurrence was the lung (50%). Pelvic recurrence developed in 11% of patients who did not receive adjuvant radiotherapy (observation or only chemotherapy), whereas none of the patients who underwent radiotherapy had a local recurrence (p=0.310). Extra-pelvic recurrence was 13% and 23.5% in patients who received chemotherapy (with or without radiotherapy) and was managed without chemotherapy (observed or underwent radiotherapy only), respectively (p=0.346). A total of 87.5% of patients with extra-abdominal recurrence did not receive chemotherapy.

The 3- and 5-year DFS were 67% and 55%, and the 3- and 5-year CSS of the entire cohort were 86% and 77%, respectively. In the univariate analysis, only age was significantly associated with DFS (p=0.022), and DFS decreased with increase in age. Menopausal status (premenopausal vs postmenopausal), tumor diameter (≤50 mm vs >50 mm), stage (IB vs IA), lymphovascular space invasion (negative vs positive), the number of lymph nodes removed (≤44 vs >44), and recurrence localization (pelvic vs extra-pelvic) were not significantly associated with DFS. There was no statistical significance for DFS between observation and receiving adjuvant therapy following staging surgery. None of the adjuvant therapies improved DFS when compared with either observation or each other. Paclitaxel-based chemotherapy, with or without radiotherapy, compared with observation or non-chemotherapy options (observation or only radiotherapy) had both 22% improvement in DFS; these differences trended toward statistical significance (p=0.079 and p=0.070, respectively). Among patients who received only adjuvant chemotherapy; although there was a 23% improvement for DFS in the paclitaxel-based chemotherapy group than in the non-paclitaxel-based group, the difference did not achieve statistical significance (86% vs 63%, p=0.126). None of the factors was significantly associated with CSS. The survival results are detailed in Table 2.

In stage IA group, there were no statistically significant improvements between observation and any adjuvant therapy or between adjuvant chemotherapy and other options without chemotherapy, for both DFS (3-year; 70% vs 80% or 75% vs 75%, p>0.05) and CSS (3-year; 88% vs 87% or 83% vs 91%, p>0.05). Stage IA disease is defined as an endometrium-confined disease with myometrial invasion occurring in less than 50% of patients. Six patients with stage IA were excluded because of missing data regarding involvement extending beyond the endometrium or not. Five patients had disease confined to the endometrium. Both 3-year DFS and CSS were 100% for patients with disease confined to the endometrium, whereas these values were 75% and 91% at the presence of myometrial invasion in stage IA (p=0.375 and p=0.594, respectively).

**Figure 1:**
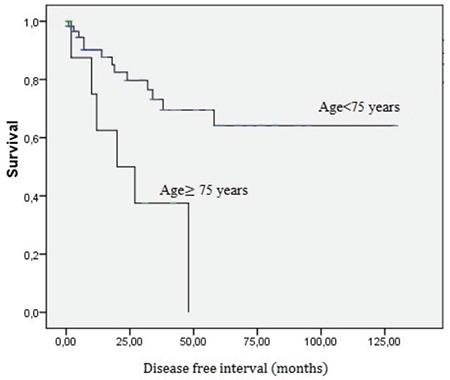
Disease-free survival decreased with increase in age.

The model of multivariate analysis for DFS included stage (stage IB vs stage IA disease), treatment (observation vs paclitaxel-based chemotherapy ± radiotherapy), and age (≥75 years vs <75 years) (Table 3). According to multivariate analysis, age was related to a statistically significant hazard ratio for a recurrence of 3.8 (95% CI=1.10-13.14, p=0.035). Advanced age was the only independent factor for recurrence (Figure 1).

## DISCUSSION

UCS has aggressive behavior with the 5-year overall survival ranging from 45% to 65% for early-stage disease (15,16). The recurrence rate varies from 30% to 50%, even if diagnosed at stage I disease (2,7). In our study, for stage I disease, the recurrence rate was 27%, the 5-year DFS was 55%, and the 5-year CSS was 77%. Similar to previous reports (2,6,7,13), distant recurrence was the most common recurrence type, in our study.

The clinical-pathologic factors that reflect both recurrence and prognosis are not apparent in early-stage disease. Deep myometrial invasion (7,17), lymphovascular space invasion presence (9), tumor size (≥5 cm) (7), history of cancer (9), older age (≥60 years) (7), and sarcoma dominance (7) are asserted as factors associated with worse survival outcomes in uterine-confined carcinosarcoma. Additionally, Leath et al. (2) reported that the only type of epithelial component (poorly differentiated endometrioid histology or serous type) was associated with an increased recurrence rate in stage I disease. In our study, advanced age was the only independent factor for DFS in stage I UCS.

Surgery is the cornerstone of UCS therapy (18,19). The necessity of adjuvant therapy is considered because of the high recurrence rate and poor survival, even in early-stage UCS. According to our knowledge, only a few studies have investigated stage I UCS, exclusively (2,6,7,8,10,13). Leath et al. (2) reported a 50% recurrence rate for patients in stage I UCS who underwent surgery alone. According to this finding, they concluded that observation is not to be considered, even in surgically staged (confirmed) patients. However, that study included a very small sample size, whose lymphadenectomy included pelvic lymphadenectomy and paraaortic sampling, with a median of nine lymph nodes removed (2). The chemotherapy-containing option, especially as chemo-radiotherapy, was claimed to be associated with better survival (6,7,13). Nonetheless, the results of these studies must be reconsidered before reaching an absolute conclusion because of the low number of lymph nodes removed.

Rauh-Hain et al. (6) analyzed the United States National Cancer Database and found that chemotherapy-containing therapy (with or without radiotherapy) was associated with improved survival compared with only surgery, among patients with stage I disease after comprehensive surgical staging. Due to the confidentiality of the database information, the technical details and adequacy of surgery could not be detailed in their report. According to Guttmann et al. (13), who studied both stages I and II UCS, chemo-radiotherapy is associated with a better overall survival than observation, radiation alone, or chemotherapy alone. Chemo-radiotherapy was linked to both improved progression-free survival and vaginal recurrence-free survival in comparison with observation, but not with radiotherapy or chemotherapy alone. Independent prognostic factors were determined as adjuvant therapy (all types of therapy vs observation) and lymphadenectomy for overall survival but only adjuvant therapy for progression-free survival and vaginal recurrence-free survival. Brachytherapy combined regimes (with chemotherapy or external beam radiotherapy) had a lower vaginal recurrence-free survival than those without brachytherapy (13). This result highlighted the potential to provide local control with low toxicity. In patients with stage I and pathologically negative nodes, Seagle et al. (8) showed that vaginal brachytherapy was accompanied by better survival, whereas adjuvant chemotherapy had no survival benefit in that cohort.

In an examination of stage 1 disease, Matsuo et al. (7) noted that chemotherapy (with or without radiotherapy) was independently concomitant with improved DFS and overall survival compared with non-use (observation or radiotherapy), both in stage I and stage IA disease. The study showed that chemotherapy was an independent predictor for both local and distant recurrence. Radiotherapy decreased local recurrence rates in the presence of risk factors, including high-grade carcinoma, sarcoma dominance, and deep myometrial invasion. Local or distant recurrence did not significantly decrease with radiotherapy or chemotherapy in patients who underwent both pelvic and paraaortic lymphadenectomy. There were no differences in DFS, overall survival, distant recurrence, and local recurrence risk between chemo-radiotherapy and chemotherapy alone (7). In contrast, Garg et al. (10) investigated elderly patients (≥65 years) with stage I UCS but found no significant improvement in survival when adding any adjuvant therapy following surgery. Similar to those of Garg et al. (10), our results showed that the addition of any adjuvant therapy neither improved DFS nor CSS, despite the relatively younger patients in our study population (median age: 65 years).

The absence of lymphadenectomy was not excluded from the eligibility criteria in the trials discussed above (6,7,10,13). This issue is important in evaluating studies because lymphadenectomy is strongly recommended, based on the presence of up to 33% of occult lymph node metastasis and the high risk of upstaging in clinically apparent uterine-confined disease (17,18,19,20). Local and distant recurrence rates significantly increased in unstaged patients, and overall survival was approximately 60% in stage I patients who were only observed postoperatively (7). Lymphadenectomy in the early stages of the disease is affiliated with an improvement in both DFS and overall survival (8,17,18). Therefore, in our study, the higher survival rate (5 year CSS=83%) in observed patients (compared with that in other studies which did not exclude lymphadenectomy), and the absence of significant differences in survival among therapy types can be attributed to the exclusion of patients with no performed lymphadenectomy and a high number of lymph nodes removed (median: 44).

Ifosfamide is accepted as the most active single agent (10). Nevertheless, combination therapies came to the forefront for improved survival. Previous studies have shown a better progression-free survival with the addition of cisplatin to ifosfamide in early-stage disease (16,21). Continued poor prognosis in UCS reflected the fact that an optimal treatment protocol has not been achieved even if stage I disease. Additionally, the high toxicity rates with limited survival advantage of ifosfamide–cisplatin regimens (21,22) have led to focusing on changing the chemotherapy procedures. In particular, clear recommendations in support of the carboplatin and paclitaxel regimen have been strongly suggested, made, especially for use in advanced stage or recurrent disease, attributed to improved survival rates with negligible toxicity rates (23,24,25,26). The effectiveness of carboplatin–paclitaxel in the early-stage is not clear. According to Guttmann et al. (13), carboplatin–paclitaxel did not affect survival when compared with other regimens in stages I/II disease. In our study, the majority (67%) of stage I patients who underwent adjuvant chemotherapy received paclitaxel-based chemotherapy (carboplatin–paclitaxel regimen). Among patients who received only adjuvant chemotherapy, although there was a 23% improvement in DFS in the paclitaxel-based chemotherapy group compared with the non-paclitaxel-based group, the difference did not achieve statistical significance. Although none of the adjuvant therapies in our results were associated with improved survival in stage I disease, DFS in the carboplatin–paclitaxel group, with or without radiotherapy, trended toward significance relative to options without chemotherapy. Nonetheless, drawing a definite conclusion is difficult because of the small sample size.

The retrospective study design and small sample size are the main limitations of this study. Data regarding the doses and the machine type used for radiotherapy could not be found from records for 24 years in all cases since the condition of delivering the radiotherapy could not be optimized. Because of the lack of consensus on standardized therapy regimens, subgroup analysis for therapy regimens is performed with a small sample size, which might potentially affect the comparisons. In our study, patients who had confirmed endometrium-confined disease with comprehensive lymphadenectomy had 100% for both DFS and CSS. This result prompted us to think that patients with endometrium-confined disease may be evaluated separately from stage IA patients. However, achieving a definitive result with such a small subgroup sample is difficult. It will be essential to assess a comparatively larger sample size with endometrium-confined UCS. Despite that, this study includes only stage I UCS cases, which were all confirmed by performing high quality, comprehensive lymphadenectomy.

Performing any adjuvant therapy following comprehensive lymphadenectomy was not linked to an improvement in survival of stage I disease. Given the still high recurrence rates in stage I UCS, further studies that include relatively larger sample numbers and a prospective design are needed to investigate therapeutic options in stage I UCS or must be the focus of new therapeutic approaches. The carboplatin–paclitaxel regimen seems to hold promise; however, drawing an accurate conclusion for early-stage disease is difficult based on current knowledge.

## Figures and Tables

**Table 1:**
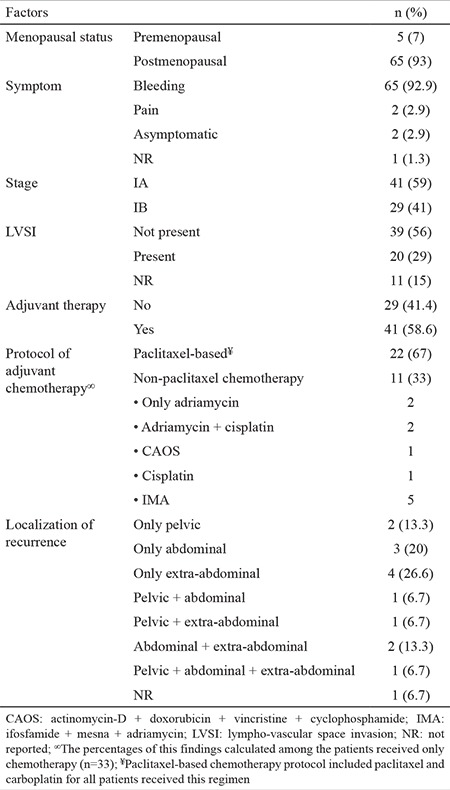
Clinical-pathological features of entire cohort

**Table 2:**
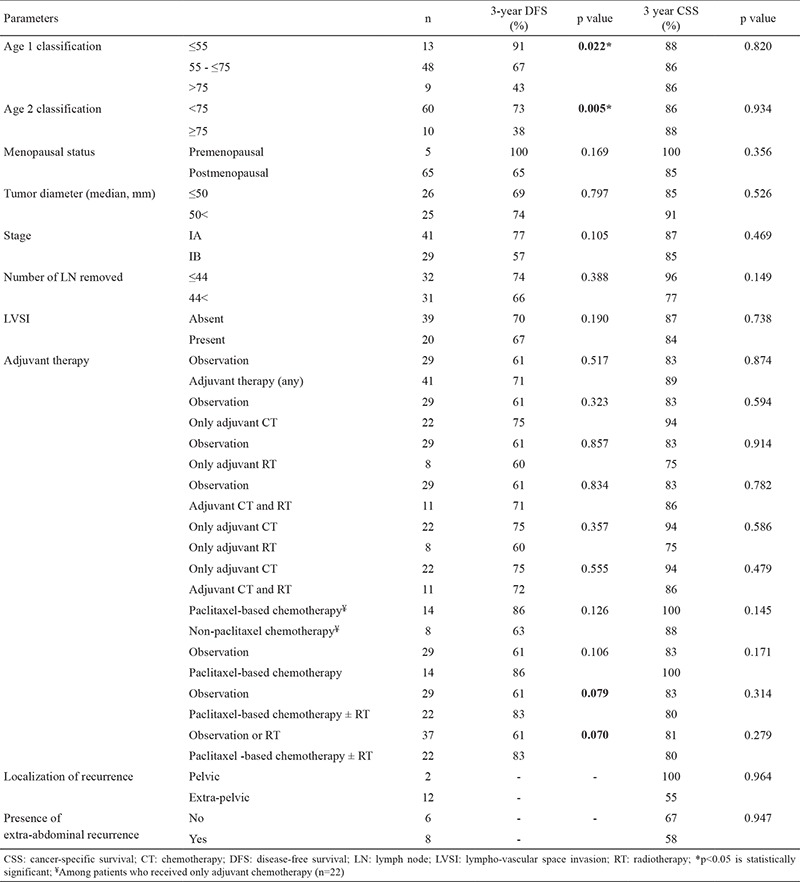
Disease-free survival and cancer-specific survival results of entire cohort

**Table 3:**
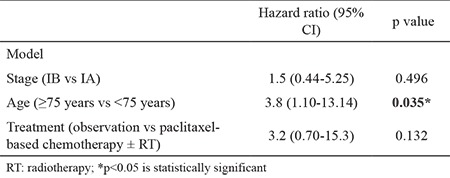
Multivariate analysis of stage I uterine carcinosarcoma for recurrence
